# Quantitative Identification of Major Depression Based on Resting-State Dynamic Functional Connectivity: A Machine Learning Approach

**DOI:** 10.3389/fnins.2020.00191

**Published:** 2020-03-27

**Authors:** Baoyu Yan, Xiaopan Xu, Mengwan Liu, Kaizhong Zheng, Jian Liu, Jianming Li, Lei Wei, Binjie Zhang, Hongbing Lu, Baojuan Li

**Affiliations:** ^1^School of Biomedical Engineering, Air Force Medical University, Xi’an, China; ^2^Network Center, Air Force Medical University, Xi’an, China

**Keywords:** sliding window, dynamic brain connectivity, static brain connectivity, resting state, machine learning

## Abstract

**Introduction:**

Developing a machine learning-based approach which could provide quantitative identification of major depressive disorder (MDD) is essential for the diagnosis and intervention of this disorder. However, the performances of traditional algorithms using static functional connectivity (SFC) measures were unsatisfactory. In the present work, we exploit the hidden information embedded in dynamic functional connectivity (DFC) and developed an accurate and objective image-based diagnosis system for MDD.

**Methods:**

MRI images were collected from 99 participants including 56 healthy controls and 43 MDD patients. DFC was calculated using a sliding-window algorithm. A non-linear support vector machine (SVM) approach was then used with the DFC matrices as features to distinguish MDD patients from healthy controls. The spatiotemporal characteristics of the most discriminative features were then investigated.

**Results:**

The area under the curve (AUC) of the SVM classifier with DFC measures reached 0.9913, while this value is only 0.8685 for the algorithm using SFC measures. Spatially, the most discriminative 28 connections distributed in the visual network (VN), somatomotor network (SMN), dorsal attention network (DAN), ventral attention network (VAN), limbic network (LN), frontoparietal network (FPN), and default mode network (DMN), etc. Notably, a large portion of these connections were associated with the FPN, DMN, and VN. Temporally, the most discriminative connections transited from the cortex to deeper regions.

**Conclusion:**

The results clearly suggested that DFC is superior to SFC and provide a reliable quantitative identification method for MDD. Our findings may furnish a better understanding of the neural mechanisms of MDD as well as improve accurate diagnosis and early intervention of this disorder.

## Introduction

Major depressive disorder (MDD) is characterized by depressed mood, lack of interest, and motivation, as well as impaired cognitive function and attention, etc. (Friedman and Anderson, 2014; Hamilton et al., 2015; Otte et al., 2016). This disorder represents a major public health issue and has been predicted to be a leading cause of disability (Wiles et al., 2013). Recent studies leveraging neuroimaging techniques have deepened our understanding of the neural mechanisms of MDD and revealed abnormalities in brain function and structure in these patients (Parlar et al., 2017). Current diagnosis of MDD is basically based on structural interview of the patients, which is expert dependent. Developing a machine learning-based approach, which could possibly realize the quantitative characterization of the brain imaging data and achieve an objective prediction of the brain disorders (Gong and He, 2015), deserves more attention.

Neuroimaging studies based on functional magnetic resonance imaging (fMRI) have provided rich evidence of abnormalities in neural activity and functional connectivity of multiple brain regions and networks of patients with MDD, including cingulate cortex, precuneus, and medial prefrontal cortex (mPFC) of the default mode network (DMN), dorsolateral prefrontal cortex (dlPFC) of the central executive network (CEN), insula of the salience network and the amygdala, hippocampus, etc. (Hamilton et al., 2015; Mulders et al., 2015; Otte et al., 2016; Ambrosi et al., 2017). These findings collectively point toward the fact that aberrant functional connectivity can be used as an imaging metric to provide new opportunities for accurate diagnosis of MDD. Likewise, recent studies used fMRI-based functional connectivity measures as eigenvalues to distinguish MDD patients from healthy subjects. Then, after leave-one-out cross validation (LOOCV), it achieved an accuracy over 70% by support vector machine (SVM) or partial least squares (PLSs) classifiers (Cao et al., 2014; Bhaumik et al., 2016; Yoshida et al., 2017).

Previous studies on resting-state functional connectivity were mainly based on the temporal correlation between regional blood oxygen level-dependent (BOLD) time courses, barring an implicit assumption that functional connectivity is temporal stationary (Sporns, 2011; Jie et al., 2018). As a matter of fact, a number of researches have revealed that functional connectivity may experience a dynamic change over time (Calhoun et al., 2014), which, to a certain extent, might be attributed to the neuronal origin and related to the cognitive and vigilance state variations (Chang et al., 2013; Thompson et al., 2013; Jie et al., 2018). By measuring time-varying functional connectivity among brain regions, dynamic functional connectivity (DFC) analysis furnishes a more detailed description of interactions in the brain. Indeed, some studies have found that the DFC analysis produced time-varying co-activation patterns, which the traditional static functional connectivity (SFC) analysis was not able to obtain (Xiao and Duyn, 2013). Thus, DFC has been applied to underlie the pathophysiology of diseases such as autism spectrum disorder (ASD), Parkinson’s disease, migraine, and seizure, etc. For example, increased dynamics of thalamic to sensory network and decreased dynamics of global network were detected in a patient with ASD (Fu et al., 2019). Clustering analysis showed that the stability of weak connection decreased while that of strong connection increased in patients with Parkinson’s disease (Kim et al., 2017), similar results were found in interictal migraine patients (Tu et al., 2019), which imply dysrhythmia in brain connections in these diseases. Besides, a high accuracy was achieved by classifying seizure patients and normal people with DFC analysis, which may help to provide a better understanding of the underlying mechanisms of this disease (Liu et al., 2017). In addition, recent research has shown that the metastable state calculated through DFC analysis was correlated to the stage of consciousness (Hudson et al., 2014; Cavanna et al., 2017), and dynamic fluctuations in functional connectivity were also suggested to be related to individual cognitive states and psychological activities (Shine et al., 2016; Pang et al., 2018).

Dynamic connectivity analysis in patients with depression has provided new insights into the neural mechanisms of this disorder. In particular, Kaiser et al. (2016) found that meditation in depressed patients is associated with abnormal communication patterns of brain fluctuations (Kaiser et al., 2016). Zhi et al. (2018) used the sliding-window algorithm to identify three types of node damage which were related to the severity of depressive symptoms and cognitive ability. Wang et al. (2019) found decreased DFC variability between the anterior DMN and the right CEN compared with controls (Wang et al., 2019). It is noteworthy that initial attempts have been made to validate that the accuracy of a machine learning-based diagnosis system could be largely improved by using DFC metrics, instead of traditional SFC measures (Zheng et al., 2019).

In the present work, we aimed to develop a machine learning-based scheme for discrimination of patients with MDD by leveraging the hidden information embedded in DFC in order to provide accurate, objective, and image-based diagnosis of MDD. Resting-state fMRI data were collected from 56 healthy controls and 43 MDD subjects. DFC was calculated using the sliding-window algorithm which is the most widely used method to investigate DFC by calculating functional connectivity in a succession of neighboring time windows (Hutchison et al., 2013a). Then, a non-linear SVM classifier-based recursive feature elimination (SVM-RFE) approach was performed to select the optimal feature subset for classification model development with a training dataset. The performance of the established model was then validated with a testing dataset and achieved a favorable accuracy and area under the curve (AUC) of receiver operating characteristic (ROC) of 0.9975. Furthermore, we investigated the spatial and temporal characteristics of the most discriminative connections. The results revealed that the most discriminative connections formed core brain networks including the frontoparietal network (FPN), visual network (VN), DMN, etc. The current study demonstrated that by combining features obtained from DFC analysis with advanced machine learning techniques, we can provide an objective and reliable image-based diagnosis system for MDD. More importantly, these findings could also provide novel insights into the underlying neural mechanisms of depression.

## Materials and Methods

### Participants

Forty-three eligible right-handed MDD patients (13 male and 30 female) were recruited from Xijing Hospital. Fifty-six healthy controls (all right-handed, 30 male and 26 female) were recruited *via* advertising. The baseline demographics of the subjects are shown in Table 1. Two-sample *t*-tests were performed to verify whether there are significant intergroup differences of age and educational level. A chi-square test was applied to verify whether the constituent ratio of gender was significant between the two groups. Statistical analyses of this study were performed by using IBM SPSS statistics (v. 22.0, Armonk, NY, United States). The level of confidence was kept at 95%, and results with *p* < 0.05 were considered significant.

**TABLE 1:** Demographics for the MDD patients and HCs.

	**HC**	**MDD**	***p*-value**
Age	32.28 ± 10.80	35.23 ± 11.23	0.157
Gender (male/female)	30/26	13/30	0.013
Educational qualifications (year)	15.78 ± 4.33	11.44 ± 3.33	<0.001
HAMD	–	23.35 ± 3.33l	–
HAMA	–	18.04 ± 3.33l	–

*Data are shown as (mean ± SD). HAMA, Hamilton Anxiety Rating Scale; HAMD, Hamilton Depression Rating Scale; HC, healthy control; MDD, major depressive disorder.*

Patients were tallied with the diagnostic criteria of Diagnostic and Statistical Manual of Mental Disorders, Fourth Edition (DSM-IV) or ASD for a current episode of MDD as assessed by two experienced psychiatrists. The severity of depression and anxiety was assessed by Hamilton Depression Rating Scale (HAMD, 24 items) and Hamilton Anxiety Rating Scale (HAMA), respectively. Exclusion criteria included incomplete HAMD test, relevant medical or neurological disorders, and incorrect head position, etc. Each of the 99 subjects was informed of the aims and procedures of the research and signed an informed consent. The experiment was carried out in strict accordance with the requirements of the Ethics Committee in Xijing Hospital.

### Acquisition and Preprocessing fMRI Data

The resting-state fMRI data were acquired at Xijing Hospital using a GE Discovery MR750 3.0 T MRI system. fMRI data were gathered from 99 subjects who completed the functional scan with the parameters set as follows: TR = 2,000 ms, TE = 30 ms, flip angle = 90°, FOV = 240 mm, matrix = 64 × 64, number of slices = 45, slice thickness = 3.5 mm, spacing = 0.0 mm. Except for the functional data, a whole-brain T1 structural image was obtained for each subject with the following parameters: TR = 8.2 ms, TE = 3.2 ms, FOV = 256 mm, matrix = 256 × 256, flip angle = 12°, slice thickness = 1 mm, no spacing.

**FIGURE 1:**
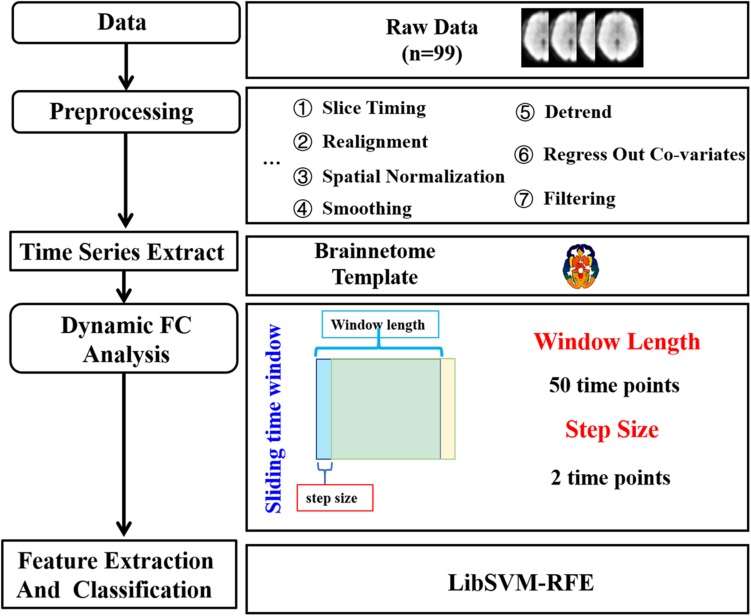
The data analysis pipeline. After data acquisition and preprocessing, sliding-window algorithm was applied with the window width set to 50 time points and the step size set to two time points to calculate dynamic functional connectivity (DFC). For each subject, a 1 × 5,653 DFC matrix was obtained after two sample *t*-test and employed as features for classification with a non-linear support vector machine (SVM) classifier.

The procedure of data analysis was shown in Figure 1. Data were preprocessed using Data Processing Assistant for Resting-State fMRI^1^ (DPARSF, version 2.3) (Yan et al., 2016). The first 10 scans of the resting-state fMRI images were discarded in order to eliminate the effects of magnetic field instability. The remaining 200 fMRI images were then corrected for slice timing, compensating the differences in acquisition time between slices. Then, realignment was performed to correct for head motion between fMRI images at different time points by translation and rotation. The high-resolution structural image was then co-registered with functional images and segmented into gray matter, white matter, and cerebrospinal fluid (CSF) signal. The deformation parameters from the structural image to the MNI template were then used to normalize the resting-state fMRI images into a standard space. Next, a Gaussian filter with a half maximum width of 6 mm was used to smooth the functional images. Then, the linear trends were removed. The effects of white matter signal, CSF signal, and Friston 24 head motion parameters were regressed out. Finally, a band-pass filter of 0.01∼0.1 Hz was used for filtering.

### Dynamic Functional Connectivity Analysis

The DFC analysis was performed by GRETNA^2^ (v2.0.0). The brain was parcellated into 274 regions according to the brainnetome atlas^3^. However, one of the regions numbered 255 with a low probability density was not identified, thus 273 regions were left. Then, Pearson’s correlation coefficient was used for measuring the functional connectivity. DFC between any pair of these regions was then calculated using a sliding-window algorithm. Sliding-window algorithm is one of the most widely used to evaluate dynamic brain functional connectivity. The functional connectivity between two nodes was first calculated using a subsection of the data within a time window. The window was then slid one step, and the calculation of the functional connectivity was repeated within the new time window. As recommended in previous studies, the window width should be no less than 1/*f*_*min*_, *f*_*min*_ represents the minimum frequency of the signal (Shakil et al., 2016; Liao et al., 2018; Guo et al., 2020). In the current study,*f*_*min*_ was 0.01 Hz and the TR was 2 s, thus the window width was set to 100 s (50 time points), and the step length was set to two time points. Finally, we obtained 76 DFC matrices for each subject, with 273 × 273 variables from each matrix.

### Feature Extraction and Selection

The upper diagonal elements of the functional connectivity matrices were extracted and 76 × 37,128 = 2,821,728 features were left for each subject to constitute the entire feature set. A linear model was used before classification to regress out the effects of gender and educational level. Then, a two-sample *t*-test (*p* < 0.001, uncorrected) was applied to select features with significant intergroup differences between the MDD patients and the health controls, resulting in a 1 × 5,635 feature vector for each subject which is the total features used in the classification. Further considering the high-throughput features extracted from the relatively limited subjects would inevitably cause redundancy and over fitting in classification, in this study, a non-linear SVM-RFE approach was employed afterward to find an optimal feature subset with the best discriminative power for MDD identification. Detailed descriptions of this widely applied approach were presented in our recent studies (Xu et al., 2019a, b).

### Performance Evaluation Using the Prediction Model Developed by the Selected Features

With the optimal feature subset selected, the prediction model was then developed for MDD identification. The non-linear SVM classifier with the radial basis function kernel was implemented using the widely used LIBSVM toolbox for model construction and performance evaluation (Chang and Lin, 2011). Labels of the patient group were set as “+1,” and that of the healthy controls were set as “–1.” The grid search approach was performed to select the optimal parameters “-c, -g” for the classification model construction. Considering the limited sample-set size, an external 10-fold cross validation (CV) strategy with 100-round classifications was used to fully evaluate the performance. This strategy first randomly and almost evenly divides the entire sample set into 10 subsets. Then, nine subsets are used to train the classifier and the remaining one subset is used to validate the trained classifier. After 10 subsets are successively validated, one round classification is finished and the average performance can be obtained. Owing to the random allocation of the 10-fold subsets, only one round classification may not well reflect the overall performance of the samples. Instead, the procedures above are usually repeated for 100 rounds, and the final average performance after all these rounds classifications can be achieved.

In order to compare the prediction performance of the current results with that of using other brain templates, the widely used Anatomical Automatic Labeling (AAL) template with 116 brain regions was adopted to repeat the steps above, including DFC, SFC feature extraction, and feature selection and classification.

Finally, the extracted DFC and SFC features were combined to classify the MDD patients from the healthy controls in both templates.

In order to verify the reliability, consistency, and generalizability of the proposed method, the database was further divided into the training set (including 73 subjects with 33 MDD patients and 40 healthy controls) and the testing set (including 26 subjects with 10 MDD patients and 16 healthy controls), accounting for about 80 and 20% of the whole datasets, respectively. The baseline demographics of the training set subjects are shown in Supplementary Table S1, the testing set subjects are shown in Supplementary Table S2. Then, a two-sample *t*-test was applied with all the features in the training set to determine the features with significant intergroup differences between MDD patients and the health controls. After that, these features with significant differences in the training set were further selected using the SVM-RFE to determine an optimal feature subset for model development. The performance of the model was then validated using the testing set.

### Relationships Between the Selected Features and Clinical Variables in the Major Depressive Disorder Group

We performed the Pearson’s correlation analysis between the selected features and clinical variables including HAMD and HAMA in the MDD group separately. Before the analysis, a linear model was used before classification to regress out the effects of gender and educational level.

## Results

### Feature Selection and Performance Evaluation

**FIGURE 2:**
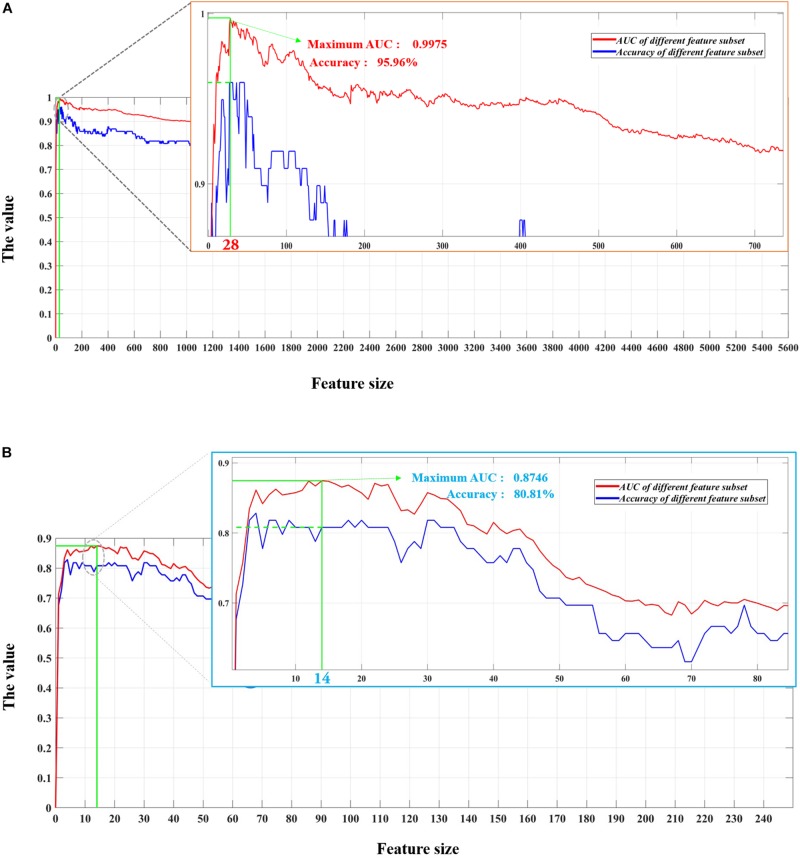
Feature selection process of using the support vector machine–classifier-based recursive feature elimination (SVM-RFE) algorithm with 5,653 dynamic functional connectivity (DFC)-based variables and 248 static functional connectivity (SFC)-based variables, respectively. Panel **(A)** represents the curve of the area under the curve (AUC) values using the top *n* features from the DFC matrices, and the red dot in the local magnification of the curve stands for the highest AUC value of 0.9975 achieved by the top 28 features. Panel **(B)** displays the curve of the AUC values using the top *n* features from the SFC matrix, and the blue dot in the local magnification of the curve shows the highest AUC value of 0.8746 achieved by the top 14 features.

Considering the imbalance of sample size between the two groups in this study, the AUC value was employed as the criterion for optimal feature subset determination (Xu et al., 2019a, b). Finally, a subset of 28 features with the highest AUC value of 0.9975 was selected as the optimal feature subset for model construction, as shown in Figure 2A.

At the same time, we compared the performances of the system using features obtained from DFC matrices with that of the system using features extracted from traditional SFC matrices. SFC matrices that measured the average functional connectivity were obtained by calculating the correlation between the whole time-series of any two nodes. Thus, for each subject, SFC analysis only resulted in one 273 × 273 functional connectivity matrix, while DFC analysis obtained 76 273 × 273 matrices. Figure 2B shows the diagnostic performances of the system with SFC matrices. Apparently, the AUC of the system was higher when using DFC matrices (0.9975) which embedded rich information on time-dependent fluctuations in connections than those using static connectivity matrices (0.8746). In addition, with the lack of features extracting time-varying connections, more (14 for static matrices compared with 28 for dynamic matrices) were required for the system to achieve its highest AUC.

**FIGURE 3:**
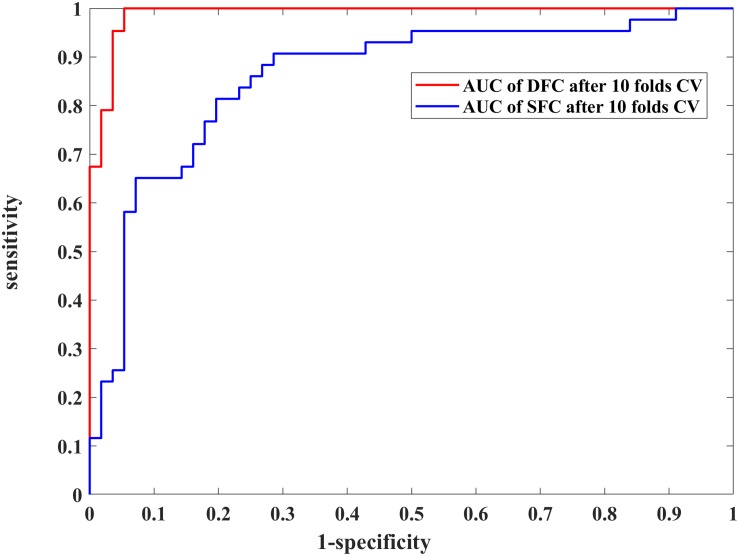
Performance comparison of the 28 selected dynamic functional connectivity (DFC)-based features and 14 selected static functional connectivity (SFC)-based features with a non-linear support vector machine (SVM) classifier and 10-fold cross validation (CV) strategy. The blue and red curves represent the receiver operating characteristic (ROC) curves of using the 28 and 14 optimal features, respectively.

We further assessed the performances of the systems with SVM and 10-fold CV (Figure 3 and Table 2). Figure 3 shows the ROCs of models constructed with the most discriminative features selected from the DFC and SFC metrics. The AUC value of the model with 28 optimal DFC features was 0.9913 while that of the model with 14 SFC features was only 0.8685. The results apparently indicated the superiority of the DFC-based classification model for MDD discrimination.

**TABLE 2:** Performance comparison between the optimal feature subsets determined from DFC and SFC using the non-linear SVM classifier and 10-fold CV with 100-round classifications.

**Method**	**Optimal features size**	**Sensitivity (%)**	**Specificity (%)**	**Accuracy (%)**	**AUC**
DFC	28	96.77	94.68	95.59	0.9913
SFC	14	76.19	83.05	80.07	0.8685

*AUC, area under the curve; CV, cross validation; DFC, dynamic functional connectivity; SFC, static functional connectivity; SVM, support vector machine.*

Then, we estimated the classification performance using the optimal feature subset selected from the DFC-based features, SFC-based features, and both of the DFC- and SFC-based features with different brain templates.

With the Brainnetome atlas template, the performance of the optimal feature subset selected from the DFC-based features in the training set was much better than that of SFC, while the performance of the optimal feature subset selected from the DFC- and SFC-based features did not witness a dramatic increase, as shown in Supplementary Figures S1, S2 and Supplementary Tables S3, S4. When using the testing data for the performance verification, we observed that the results of the DFC-based optimal features were also the best, whereas the performance of the other two optimal feature subsets received a dramatic decline, indicating the low consistency and generalizability of the models developed by using these two optimal feature subsets.

With the AAL atlas template, the performance of the optimal feature subset selected from the DFC-based features in the training set was also much better than that of SFC, while the performance of the optimal feature subset selected from of the DFC- and SFC-based features was nearly the same with that of the DFC-based optimal features, as shown in Supplementary Figure S3 and Supplementary Table S5. When using the testing data for verification, we also noticed that the performance of the DFC-based optimal features was the best but was severely inconsistent with the performance of using these optimal features in the training set, as shown in Supplementary Figure S4 and Supplementary Table S6.

All the results above conclusively reveal that the classification model developed using the DFC-based optimal features extracted from the brain regions using the Brainnetome atlas template could be more powerful for the discrimination between the MDD patients and the healthy people; the SFC-based features could probably introduce certain feature redundancy that might further impair the discriminative power of the prediction model.

### Spatiotemporal Characteristics of the Most Discriminative Dynamic Functional Connections

**FIGURE 4:**
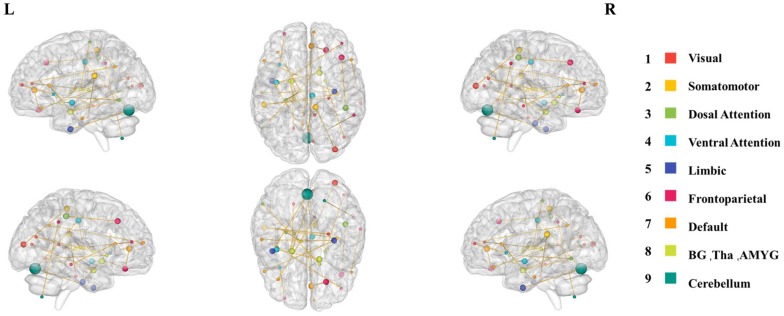
The spatial distribution of the 28 most discriminative dynamic connections after feature selection. The size of the node represents the node degree, while the color of the node represents the brain network that this node belongs to.

**FIGURE 5:**
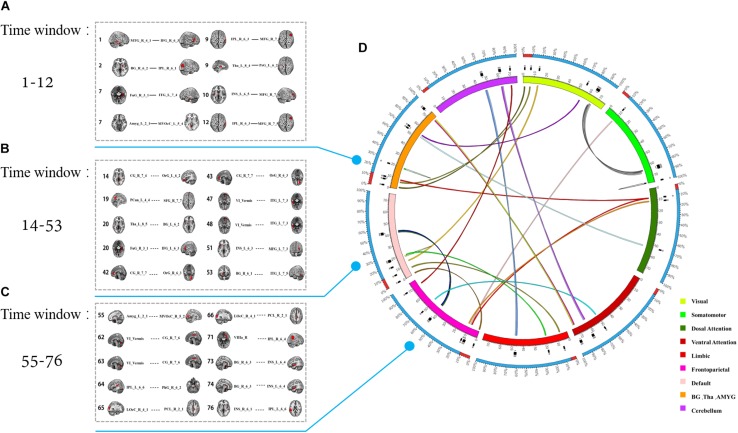
The temporal distribution of the 28 most discriminative dynamic connections after feature selection. **(A–C)** The connections in different time windows. **(D)** The color of the inner circle represents the brain network that the node belongs to. The lines in the circle represent the connections, and the color of the connections represents different windows.

In fact, the 28 most discriminative DFC connections were 28 abnormal DFCs in patients with MDD compared with healthy controls. We further investigated the spatiotemporal characteristics of the most discriminative dynamic functional connections in patients with MDD. More specifically, the 28 abnormal DFCs involved seven different brain networks and 40 different brain regions, distributed in 24 sliding windows. Figure 4 shows the spatial pattern of these connections which clearly suggested that these connections form brain networks including the VN, somatomotor network (SMN), dorsal attention network (DAN), ventral attention network (VAN), limbic network (LN), FPN, and DMN, etc. Table 3 provided more detailed information on these connections with their discriminative power (weight). According to Table 3, the connections that demonstrated the highest discriminative power included DFC connections between the inferior parietal lobule (IPL) and middle frontal gyrus (MFG), between parahippocampal gyrus and IPL, between cingulate gyrus and orbital gyrus. Figure 5 shows the temporal characteristics of these 28 connections. The most discriminative connections within each time window were depicted. We found that over time, the brain area gradually penetrated from the cortex to the deeper regions of the brain. More importantly, Figure 5 also implicated that the most discriminative connections varied largely from one time window to another. Thus, traditional SFC analysis which is unable to capture the time-dependent variations in functional connections would fail to detect these most discriminative connections.

**TABLE 3:** The 28-dynamic function connectivities.

**Number**	**Functional connectivity**	**Sliding time window**	**Weight**
(1)	IPL_R_6_3 MFG_R_7_5	9	1.00
(2)	IPL_L_6_6 PhG_R_6_2	64	0.96
(3)	CG_R_7_4 OrG_L_6_2	14	0.93
(4)	BG_R_6_3 INS_L_6_4	74	0.89
(5)	VI_Vermis ITG_L_7_3	47	0.85
(6)	LOcC_R_4_1 PCL_R_2_1	65	0.81
(7)	Tha_L_8_5 BG_L_6_2	20	0.78
(8)	FuG_R_3_1 ITG_L_7_4	7	0.74
(9)	INS_L_6_5 MFG_R_7_4	10	0.70
(10)	VIIIa_R IPL_R_6_4	71	0.67
(11)	INS_L_6_3 MFG_L_7_3	51	0.63
(12)	INS_R_6_1 IPL_L_6_6	76	0.59
(13)	CG_R_7_7 OrG_R_6_3	42	0.56
(14)	BG_R_6_2 IPL_R_6_1	2	0.52
(15)	Tha_L_8_4 PrG_L_6_2	9	0.48
(16)	BG_R_6_1 ITG_L_7_5	53	0.44
(17)	VI_Vermis CG_R_7_6	63	0.41
(18)	LOcC_R_4_1 PCL_R_2_1	66	0.37
(19)	MTG_R_4_1 IFG_R_6_3	1	0.33
(20)	VI_Vermis ITG_L_7_3	48	0.30
(21)	Amyg_L_2_1 MVOcC_L_5_4	7	0.26
(22)	FuG_R_3_1 IFG_L_6_3	20	0.22
(23)	VI_Vermis CG_R_7_6	62	0.19
(24)	CG_R_7_7 OrG_R_6_3	43	0.15
(25)	IPL_R_6_3 MFG_R_7_5	12	0.11
(26)	Amyg_L_2_1 MVOcC_R_5_2	55	0.07
(27)	PCun_L_4_4 SFG_R_7_7	19	0.04
(28)	BG_R_6_3 INS_L_6_4	73	0.00

### Relationship With Clinical Properties

**FIGURE 6:**
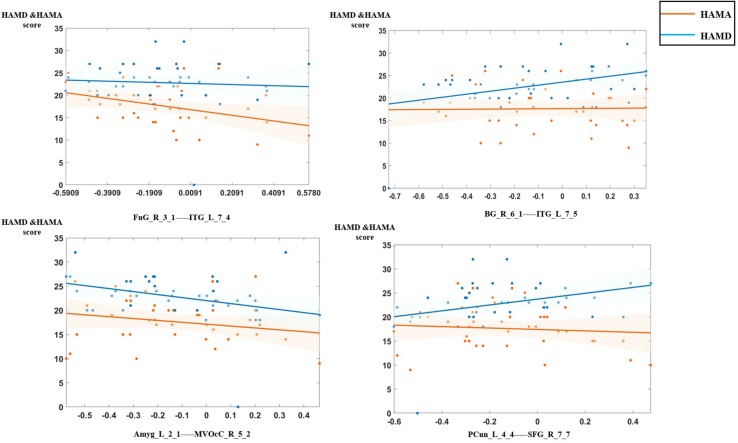
The relationship between the optimal dynamic functional connectivity (DFC) features and clinical characteristics in the major depressive disorder (MDD) group.

In the analysis of correlations between the selected features and clinical characteristics in the MDD group, we found that the dynamic functional connection between fusiform gyrus and inferior temporal gyrus was significantly negatively correlated with HAMD scores, connections between basal ganglia and inferior temporal gyrus, the precuneus and superior frontal gyrus (SFG) were significantly positively correlated with HAMA scores, connection between medioventral occipital cortex and the amygdala was significantly negatively correlated with HAMA scores. Figure 6 shows the details of relationships between DFC features and clinical characteristics.

## Discussion

In this study, we developed a machine learning diagnosis framework for patients with MDD based on subjects’ dynamic resting-state functional connectivity patterns. Three main findings emerged from the current study: (1) Patients with MDD could be reliably differentiated from healthy controls based on the patterns of resting-state DFC with a high accuracy of 0.9913 (10-fold CV). (2) Spatially, the most discriminative connections formed core networks including the VN, SMN, DAN, VAN, LN, FPN, and DMN, etc. (3) Temporally, the most discriminative connections were not stationary as assumed by traditional SFC analysis. On the contrary, these connections varied from the cortex to deeper structures of the brain over time.

### Static Functional Connectivity Analysis Versus Dynamic Functional Connectivity Analysis

Although the high-throughput feature set containing 5,635 variables were obtained from the upper diagonal elements of the DFC matrices, they might not contribute equally for the distinction between MDD patients and the healthy controls. In fact, the features highly correlated with each other or less capable of MDD identification would inevitably cause redundancy for the classifier training, impairing the overall discriminative power for MDD patients. Therefore, the SVM-RFE approach was employed to determine an optimal feature subset for prediction model construction (Xu et al., 2019a, b). The classification performance of the model using all the 5,635 variables and a non-linear SVM classifier with LOOCV achieved the sensitivity, specificity, accuracy, and AUC of 53.49, 71.43, 63.64%, and 0.7072, respectively, whereas these metrics were greatly improved to 97.67, 94.64, 95.96%, and 0.9975 using the model constructed by the 28 optimal features and the same classifier with LOOCV. It apparently demonstrates the effectiveness and great potential of SVM-RFE approach for redundancy reduction, optimal feature determination, and performance improvement. Concerning the LOOCV might introduce the overtraining in the classification, the non-linear SVM classifier with 10-fold CV was further employed to evaluate the performance of the optimal features. The results demonstrate the favorable robustness and consistency of the model for MDD diagnosis.

As far as we know, most of the previous studies were based on the resting-state functional connectivity, and the accuracy of the distinction between MDD patients and the healthy controls varied between 76.10 and 91.90% (Bhaumik et al., 2017; Li et al., 2017; Yoshida et al., 2017; Zhong et al., 2017). However, a growing number of studies suggest that resting-state functional connectivity may hide some information, which could be fully reflected in DFC (Zhang et al., 2019; Zheng et al., 2019). In order to compare the capability of the DFC and SFC matrices for the quantitative characterization of patients with MDD, the prediction model using the optimal features extracted from the SFC matrix was also developed. The classification performance was apparently inferior to that of the DFC-based prediction model, denoting that the DFC might effectively describe the network changes associated with the feelings and executive function that closely relate to MDD, thus could obtain more excellent classification performance when used for MDD identification (Zhang et al., 2019).

The potential explanation of the superiority of the DFC-based prediction model is that during the resting-state scanning, subjects were required to simply close their eyes without thinking about anything. However, there still exist mind wandering and attention return. These cognitive processes may lead to huge fluctuations of brain connections during scanning (Chang and Glover, 2010). Such time-varying information of the spontaneous brain activity could not be reflected in SFC but might be captured by DFC using the sliding-window algorithm with appropriate window width and step size.

Besides, the results of the DFC-based prediction model further suggest that the present method could not only improve the classification performance in comparison with state-of-the-art approaches (Demirtas et al., 2016) but also shed light upon the temporal patterns of brain activity and their applications in brain disorder diagnoses.

### Spatial Characteristics of the Most Discriminative Connections

Using the SVM-RFE approach, we selected 28 optimal features from 5,635 dynamic functional connections obtained in 76 time windows. The spatiotemporal characteristics of these most discriminative connections were then investigated by mapping these connections into the 76 DFC matrices to reconstruct the spatial patterns and analyze the temporal characteristics of these connections. Interestingly, we noted that although several brain networks were implicated, a large portion of these connections were associated with the FPN, VN, and DMN. The findings indicated that these regions contributed largely to accurate classification of MDD patients with healthy controls and thus may play an important role in the neural mechanisms of MDD.

The most discriminative connections formed several core brain networks including the VN, SMN, DAN, VAN, LN, FPN, and DMN, etc., suggesting that connectivity of these networks may be disrupted in patients with MDD. The results are in line with numerous previous studies that have observed abnormal connectivity of these networks (Wang et al., 2012; Wu et al., 2013, 2017; Hilland et al., 2018; Fan et al., 2019; Yu et al., 2019). Among these networks, the connection between MFG and IPL demonstrated the most significant contribution to the accurate classification of MDD patients with healthy controls. Previous researchers found that MFG and SFG showed decreased functional connectivity in MDD with robustness (Sheng et al., 2018; Yang et al., 2019). Cui et al. (2018) found that the global functional connectivity of the right IPL increased in MDD patients compared with the control group in two distinct datasets, and IPL is one of the discriminative effective connections when distinguishing MDD patients and healthy controls in a classification study with an accuracy of 91.67% (Geng et al., 2018). More importantly, both of the MFG and IPL are subregions of FPN, which is a cognitive control network, especially a goal-directed regulation of attention and emotion, etc. (Marek and Dosenbach, 2018). Leming et al. (2019) found disruption of normative pathways in FPN in MDD, and others found abnormal connections between FPN and some networks such as DMN in MDD (Disner et al., 2011). These results were consistent with those of a cognitive model that disorders of goal-directed attention and emotion can lead to excessive rumination (Kaiser et al., 2015). Besides, another study also reported that three subnetworks in the FPN of MDD patients had increased functional connectivity before treatment and recovered after treatment, which makes it a potential target for antidepressant therapy (He Y. et al., 2018).

Default mode network is a central network for MDD which was verified in numerous researches in the last decades, most of results contribute to a conclusion that the aberrant function and structure of it was related to depressive rumination. Kühn et al. (2012) found that rumination was correlated negatively with the volume of gray matter in the anterior cingulate cortex and other regions. In the functional connectivity analysis, a meta-analytic result showed an increased connection between DMN and subgenual prefrontal cortex, which was able to predict rumination level (Hamilton et al., 2015). Besides, a DFC analysis was conducted by Kaiser et al. (2016), and they reported that the increased DFC between the mPFC and the insula was correlated with the level of rumination. However, a recent study applied a multicenter research with 1,642 participants and reached a conclusion that reduced but not increased connection was only in recurrent MDD, and this had a positive relation with symptom severity (Yan et al., 2019). Our result also showed that the DFC between the precuneus and SFG was significantly positively correlated with HAMA scores.

VN was also an important region that contributed to classification. Our findings are consistent with recent studies that have noticed functional and structural abnormalities in the VN in MDD patients. Zeng et al. (2012) used SFC analysis to classify patients with MDD and healthy controls, and they reported that the VN was among the most discriminative regions (Zeng et al., 2012). Latterly, in the study of functional connectivity density (FCD), a decrease in long-term FCD of supraoccipital gyrus was found, suggesting that the visual cortex is a key hub for MDD (Zou et al., 2016). In addition, the structure of the VN was also impaired in the patients. Significantly thinner calcarine gyrus was found in MDD patients than in healthy controls (Suh et al., 2019). Occipital bending is a powerful biomarker for depression, and patients with occipital bending were reported to have abnormal cortical thickness in the posterior occipital lobe (Fullard et al., 2019). Notably, a recent study revealed that brain regions associated with early awakening and visual processing overlap in patients with MDD (Tao et al., 2018). Thus, impairments in the visual areas may result in disruption of sleep rhythms and symptoms of sleep disturbances generally seen in patients with MDD.

When analyzing the temporal characteristics of the most discriminative features, we found that these connections were distributed in different time windows, reflecting the non-stationary characteristics of functional connectivity over time which has been consistently noticed in literature (Xiao and Duyn, 2013; Bi et al., 2016; Demirtas et al., 2016; Kaiser et al., 2016; Du et al., 2018; He C. et al., 2018). These results thus provide an explanation why the classification model with the DFC features could achieve better performances than the model with SFC features, since traditional static analysis may eliminate the contribution of the more volatile connections (Britz et al., 2010; Chang and Glover, 2010; Hutchison et al., 2013b). Furthermore, we noticed that the most discriminative connections gradually changed from cortical regions to deeper structures of the brain over time, suggesting a switch between the cortical and limbic systems in the patients at rest.

To summarize, the current study used a data-driven machine learning approach to demonstrate that by leveraging valuable information embedded in DFC metrics, we could provide an accurate diagnosis scheme for patients with MDD. The spatiotemporal characteristics of those most discriminative connections could provide a novel insight into the neural mechanisms of this disorder.

### Limitations

This study has some limitations and caveats to bear in mind. Although sliding-window algorithm is one of the most widely used methods to investigate DFC, a recent study has suggested that this algorithm tends to suppress dynamic correlation, especially those that change rapidly with time (Mokhtari et al., 2019). In addition, the step size and window width should be carefully set for the sliding-window algorithm. For the current study, we set the window width to 50 time points and the step size to two time points as suggested by previous studies (Guo et al., 2020). We will further investigate the effects of different parameter settings in future studies. Finally, we used machine learning to successfully distinguish depression patients from normal people. However, the sample size in the current study is relatively small. Future studies may independently replicate our results on large sample datasets. Despite many limitations, our study suggested that by combining dynamic resting-state functional connectivity analysis and machine learning techniques, we were able to provide a reliable imaging-based quantitative identification of major depression for early intervention in MDD patients.

## Data Availability Statement

The datasets generated for this study are available on request to the corresponding author.

## Ethics Statement

The studies involving human participants were reviewed and approved by the Ethics Committee in Xijing Hospital. The patients/participants provided their written informed consent to participate in this study.

## Author Contributions

BL, XX, and HL designed the study and provided critical suggestion for the procedure. BY, ML, KZ, JLiu, JLi and LW collected and collated the data. KZ, BY, XX, LW, and BZ conducted the data analysis and helped to interpret the results. XX, ML, BY, and BL drafted the manuscript. All authors thoroughly reviewed the manuscript with no confliction of the content and signature.

## Conflict of Interest

The authors declare that the research was conducted in the absence of any commercial or financial relationships that could be construed as a potential conflict of interest.

## Footnotes

1 http://www.rfmri.org/DPARSF

2 https://www.nitrc.org/projects/Gretna

3 http://atlas.brainnetome.org/
