# Supplementary material for: Quantitative Identification of Major Depression Based on Resting-State Dynamic Functional Connectivity: A Machine Learning Approach
Source: Front Neurosci. 2020 Mar 27;14:191. doi: 10.3389/fnins.2020.00191 (PMC7118554; doi:10.3389/fnins.2020.00191)

# Supplementary Materials

Fig.S1 Feature selection results of using the statistically significant features with SVM-RFE approach in the training set: (A) 20 optimal features selected using the DFC-based features with statistical significance; (B) six optimal features selected using the SFC-based features with statistical significance; (C) 13 optimal features selected using both the DFC- and SFC-based features with statistical significance.

**(A)**

**
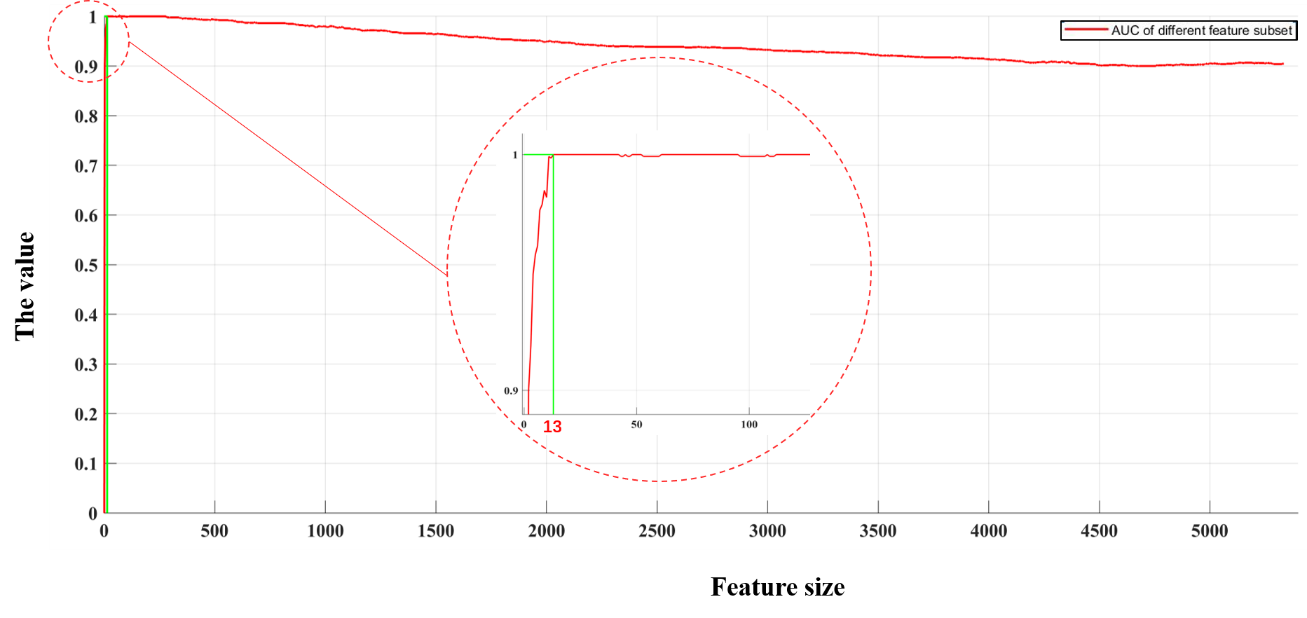
**

**(B)**

**
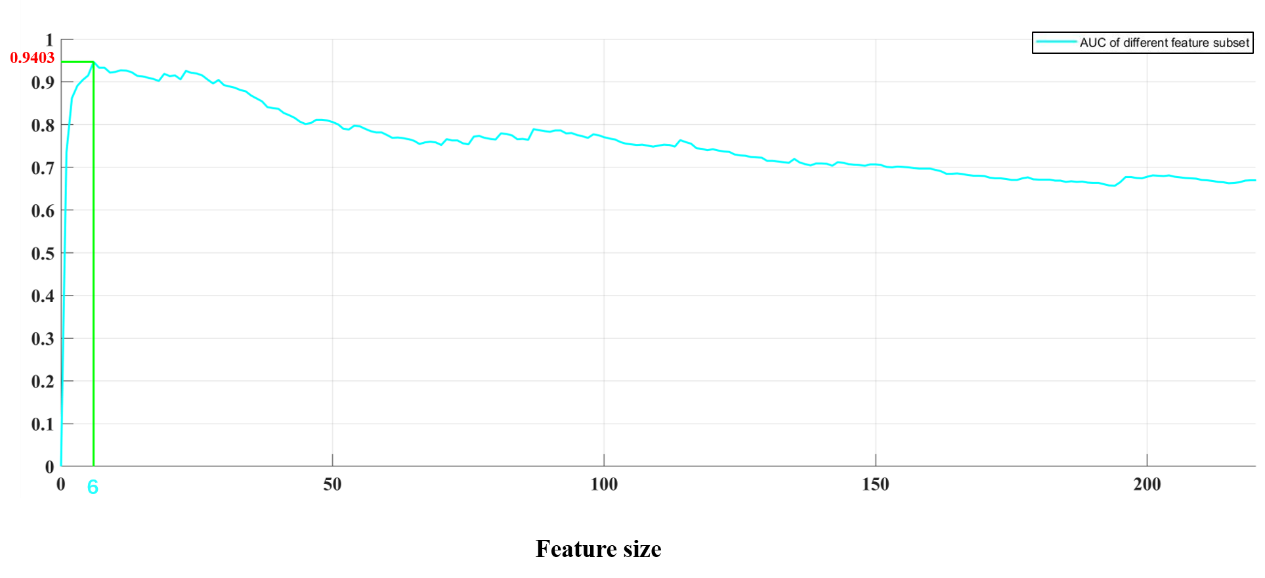
**

**(C)**

**
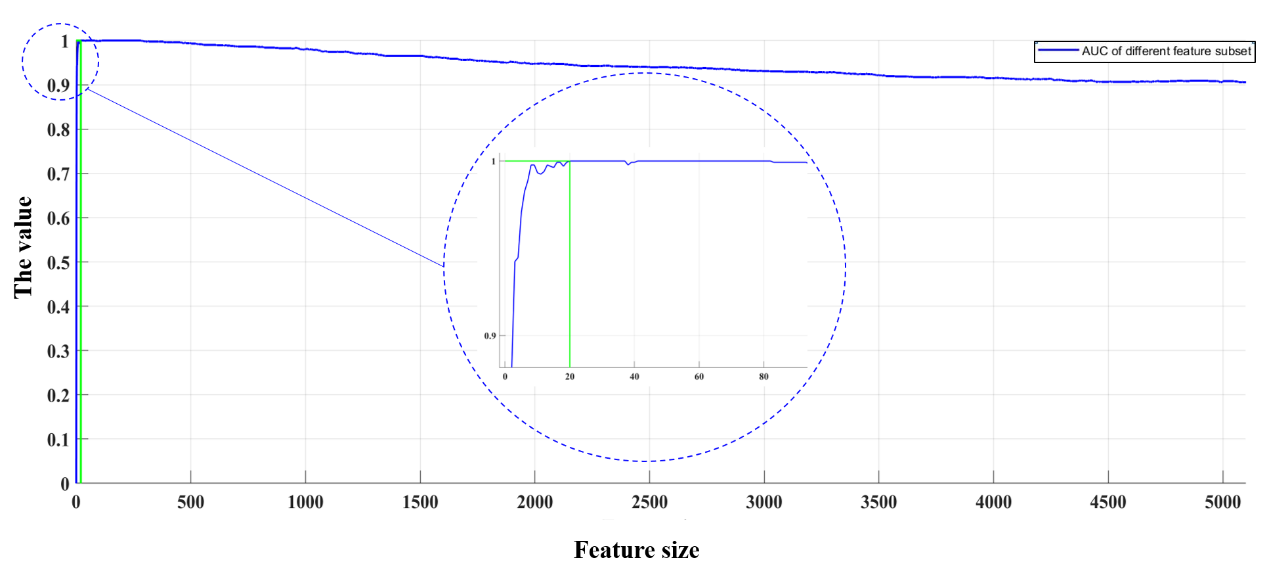
**

Fig. S2 the ROC curves of using the optimal models with the testing set


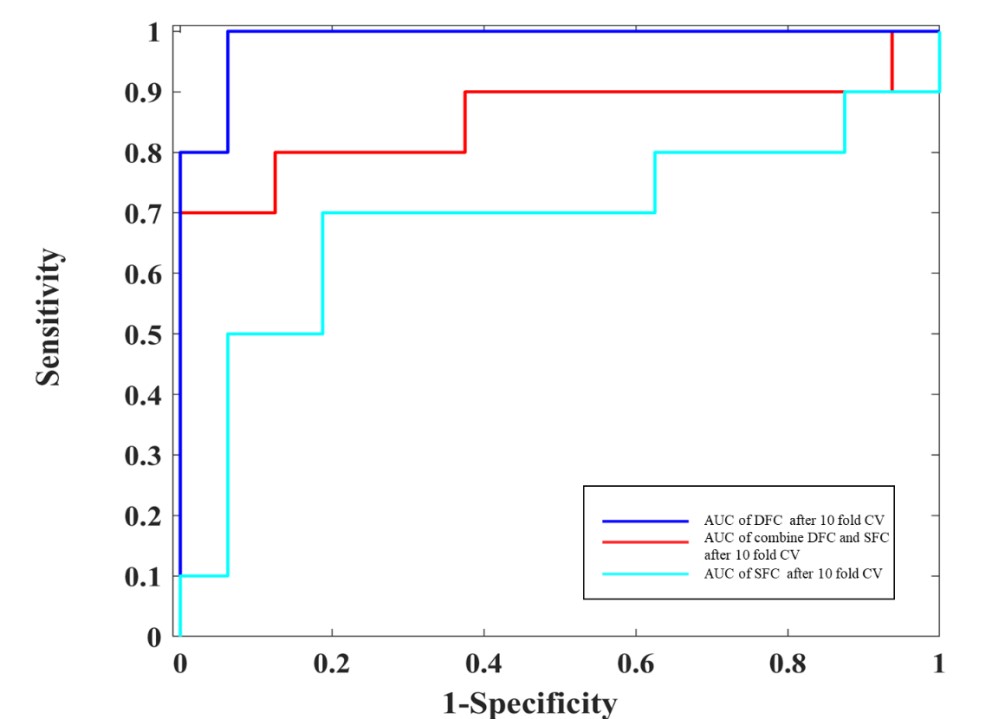


Figure S3 Feature selection results of using the statistically significant features with SVM-RFE approach in the training set: (A) 12 optimal features selected using the DFC-based features with statistical significance; (B) five optimal features selected using the SFC-based features with statistical significance; (C) 14 optimal features selected using both the DFC- and SFC-based features with statistical significance.

**(A)**

**
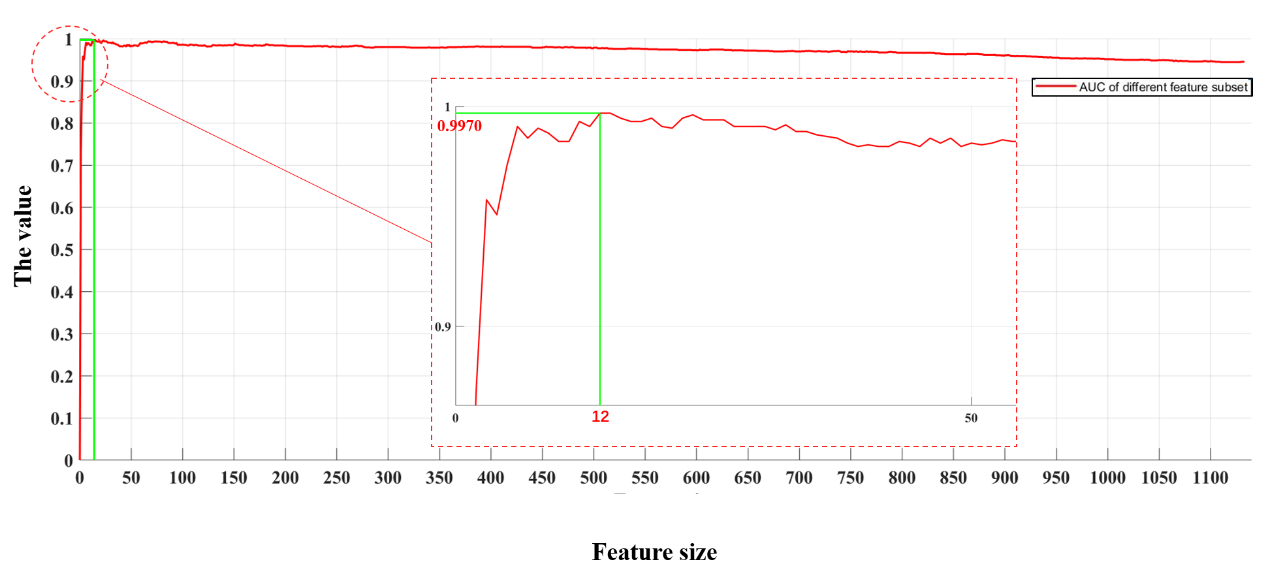
**

**(B)**

**
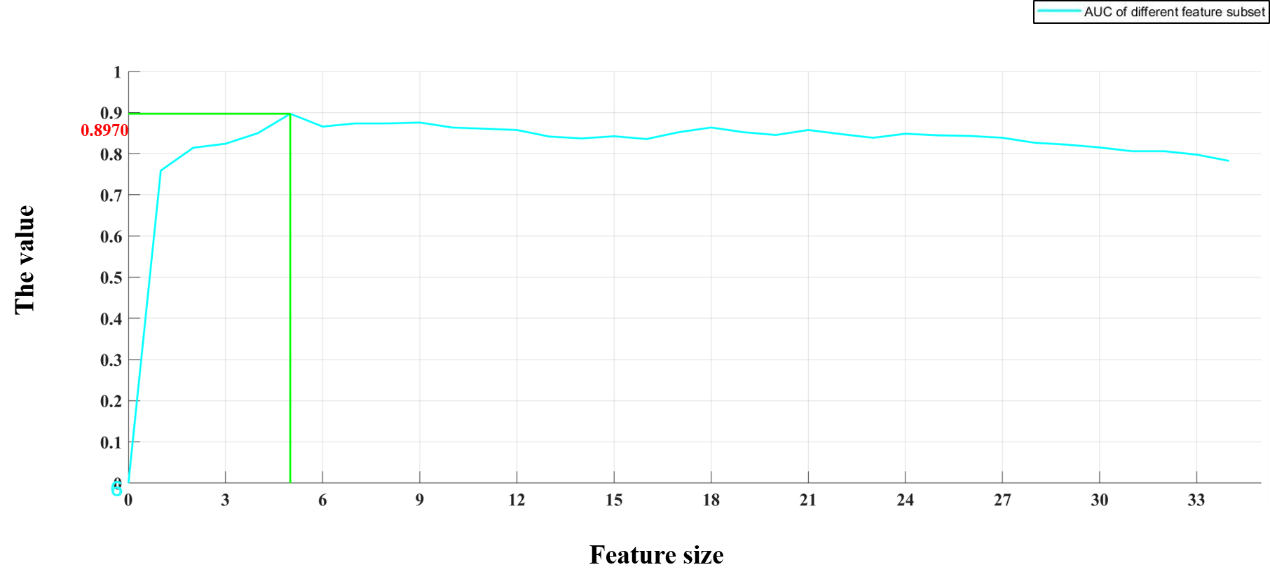
**

**(C)**


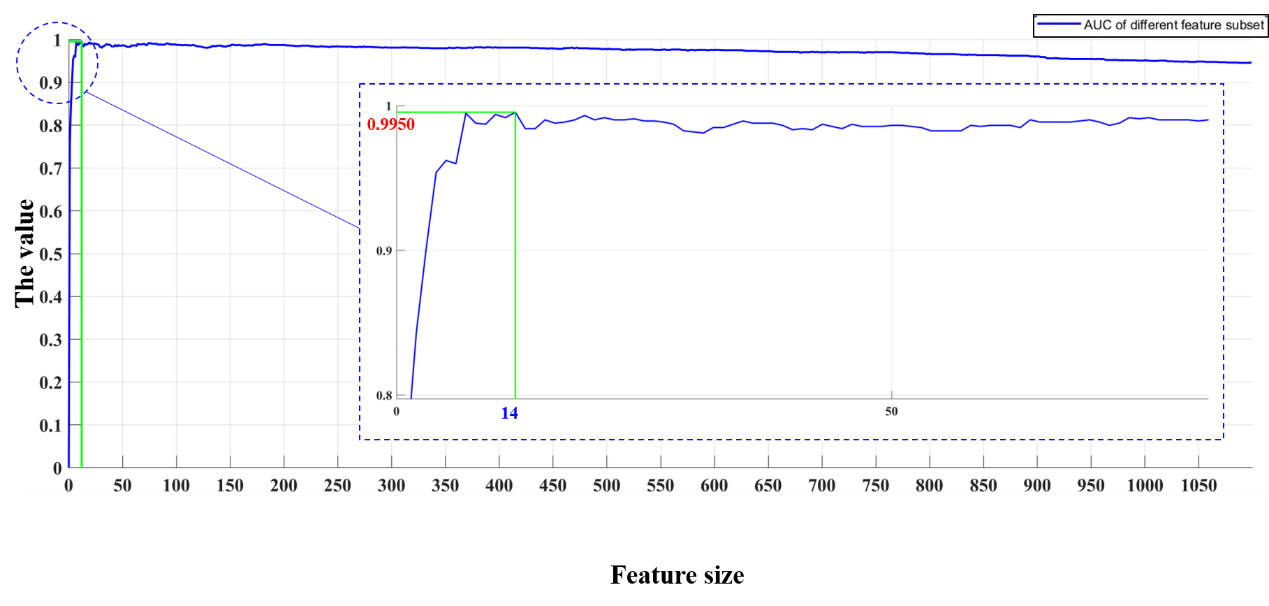


Fig. S4 the ROC curves of using the optimal models with the testing set


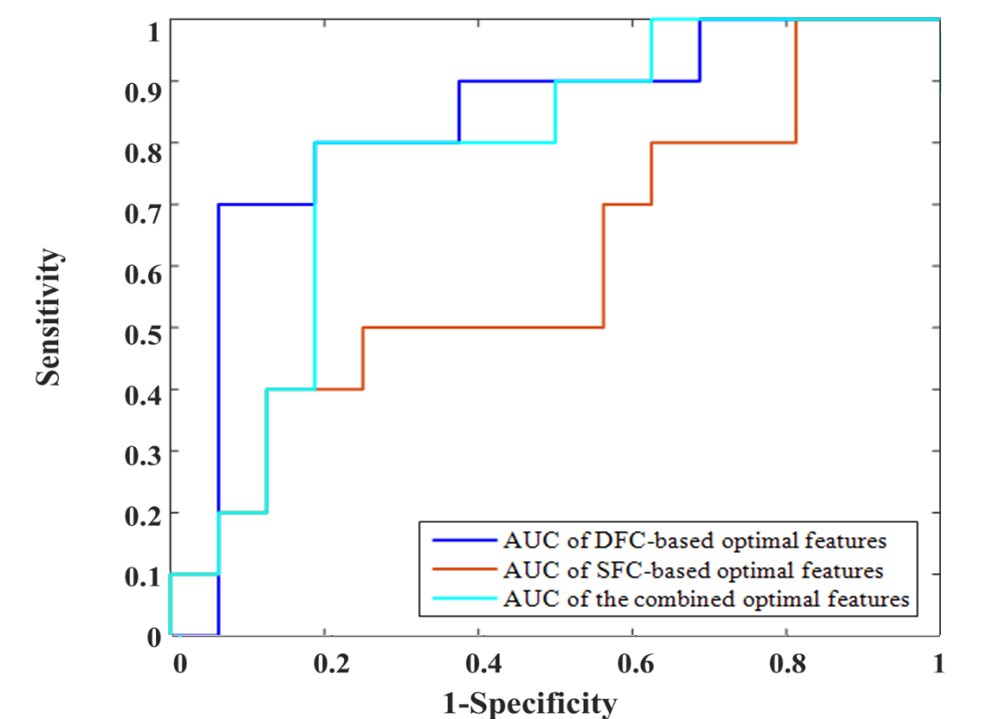

Supplement: Supplementary file 1 [file Data_Sheet_1.docx]
