# Supplementary material for: Quantitative Identification of Major Depression Based on Resting-State Dynamic Functional Connectivity: A Machine Learning Approach
Source: Front Neurosci. 2020 Mar 27;14:191. doi: 10.3389/fnins.2020.00191 (PMC7118554; doi:10.3389/fnins.2020.00191)
Supplement: Supplementary file 2 [file Table_1.docx]

# Supplementary Materials

## I. Allocation of the training and testing sets

The database was randomly divided into the training and testing sets to develop the prediction model and verify its performance. The training set contained 40 healthy controls (HC) and 33 MDD patients, and the testing set consisted of 16 HC and 10 MDD patients.

**Table S1: Demographics for the training feature subsets of MDD patients and healthy controls (HC)**

|  | HC | MDD | p-value |
| --- | --- | --- | --- |
| Number | 40 | 33 | **-** |
| Age | 32.22±11.28 | 37.66±10.65 | 0.039 |
| Gender (male / female) | 24/16 | 7/26 | <0.001 |
| Educational Qualifications (year) | 16.02±4.43 | 10.96±3.31 | <0.0001 |
| HAMD | **-** | 17.90±4.49 | *-* |
| HAMA | - | 23.45±3.45 | - |

Data are shown as (mean ± SD)

**Table S2: Demographics for the testing feature subsets of MDD patients and the HC**

|  | HC | MDD | p-value |
| --- | --- | --- | --- |
| Number | 16 | 10 | **-** |
| Age | 32.00±10.00 | 27.20±9.56 | 0.238 |
| Gender (male / female) | 6/10 | 6/4 | 0.078 |
| Educational Qualifications (year) | 15.18±4.16 | 13.00±3.05 | 0.281 |
| HAMD | **-** | 16.70±7.27 | **-** |
| HAMA | - | 20.70±7.83 | - |

Data are shown as (mean ± SD)

## II. The performance of the classification model developed by the training set and validated by the testing set with the features extracted based on Brainnetome atlas

After feature extraction based on Brainnetome atlas, the feature selection process was implemented subsequently using Student’s *t*-test, first, to select the features with significant inter-group differences between the HC and MDD patients in the training set. As for the DFC-based, SFC-based, and both DFC- and SFC-based features, 5100, 234, and 5334 features with statistically significant differences were selected, respectively. Then, these features were further selected using the SVM-RFE algorithm in the training sets based on the 5100 DFC-based, 234 SFC-based , and 5334 of both DFC- and SFC-based features with statistical significance, respectively.

Fig.S1 Feature selection results of using the statistically significant features with SVM-RFE approach in the training set: (A) 20 optimal features selected using the DFC-based features with statistical significance; (B) six optimal features selected using the SFC-based features with statistical significance; (C) 13 optimal features selected using both the DFC- and SFC-based features with statistical significance.

From Fig. S1, we can find that 20 optimal DFC-based features, 6 optimal SFC-based features and 13 optimal features with both the DFC- and SFC-based features were determined as the optimal feature subsets in the training set based on the criteria of the maximum AUC, respectively. The performance of using the three optimal feature subsets with the training set for the classification of MDD and HC using the nonlinear SVM classifier and 10- fold CV with 100-round classifications was achieved, as shown in Table S3.

**Table S3. Performance comparison between the optimal feature subsets determined from DFC, SFC, and both of DFC and SFC using the nonlinear SVM classifier and 10- fold CV with 100-round classifications in the training set**

| Method | Optimal features size | Sensitivity | Specificity | Accuracy | AUC |
| --- | --- | --- | --- | --- | --- |
| DFC | 20 | 100% | 97.47% | 98.62% | 0.9997 |
| SFC | 6 | 87.18% | 91.22% | 89.40% | 0.9403 |
|  |  |  |  |  |  |
| Combine | 13 | 100% | 98.57% | 99.21% | 1.0000 |

Using the models developed by the optimal feature subsets with the training set, we finally obtained the verification results using the testing set, respectively, as shown in Table S4.

**TableS4:** **Performance comparison between the optimal feature subsets determined from DFC, SFC, and both of DFC and SFC using the nonlinear SVM classifier and 10- fold CV with 100-round classifications in the testing set**

| Method | Optimal features size | Sensitivity | Specificity | Accuracy | AUC |
| --- | --- | --- | --- | --- | --- |
| DFC | 20 | 88.60% | 93.13% | 91.38% | 0.9771 |
| SFC | 6 | 62.7% | 71.50% | 68.12% | 0.6597 |
|  |  |  |  |  |  |
| Combine | 13 | 80.00% | 87.50% | 84.62% | 0.8563 |

The results of using the classification models with the testing set showed that DFC-based optimal features achieved the best performance, which was also very consistent with the performance using the training set. Figure S2 shows the ROC curves of using the optimal models with the testing set.

Fig. S2 the ROC curves of using the optimal models with the testing set

## III. The performance of the classification model developed by the training set and validated by the testing set with the features extracted based on AAL atlas (116 brain regions)

We further evaluated the AAL atlas-based features extracted from 116 brain regions for the discrimination of MDD patients.

After feature extraction, the feature selection process was implemented subsequently using Student’s *t*-test, first, to select the features with significant inter-group differences between the HC and MDD patients in the training set. As for the DFC-based, SFC-based, and both DFC- and SFC-based features, 1099, 34, and 1133 features with statistically significant differences were selected, respectively. Then, these features were further selected using the SVM-RFE algorithm in the training sets, respectively. The selection process is exhibited in Fig. S3.

Figure S3 Feature selection results of using the statistically significant features with SVM-RFE approach in the training set: (A) 12 optimal features selected using the DFC-based features with statistical significance; (B) five optimal features selected using the SFC-based features with statistical significance; (C) 14 optimal features selected using both the DFC- and SFC-based features with statistical significance.

From Fig. S3, we can find that 12 optimal DFC-based features, 5 optimal SFC-based features and 14 optimal features with both the DFC- and SFC-based features were determined as the optimal feature subsets in the training set based on the criteria of the maximum AUC, respectively. The performance of using the three optimal feature subsets with the training set for the classification of MDD and HC using the nonlinear SVM classifier and 10- fold CV with 100-round classifications was achieved, as shown in Table S5.

**Table S5: Performance comparison between the optimal feature subsets determined from DFC, SFC, and both of the DFC and SFC features based on AAL atlas using the nonlinear SVM classifier and 10- fold CV with 100-round classifications in the training set**

| Method | Optimal features size | Sensitivity | Specificity | Accuracy | AUC |
| --- | --- | --- | --- | --- | --- |
| DFC | 12 | 93.94% | 97.50% | 95.89% | 0.9955 |
| SFC | 5 | 69.70% | 87.50% | 79.45% | 0.8970 |
|  |  |  |  |  |  |
| Combine | 14 | 93.94% | 97.50% | 95.89% | 0.9970 |

Using the models developed by the optimal feature subsets with the training set, we finally obtained the verification results using the testing set, respectively, as shown in Table S6.

**Table S6: Performance comparison between the optimal feature subsets determined from DFC, SFC, and both of DFC and SFC features based on AAL atlas using the nonlinear SVM classifier and 10- fold CV with 100-round classifications in the testing set**

| Method | Optimal features size | Sensitivity | Specificity | Accuracy | AUC |
| --- | --- | --- | --- | --- | --- |
| DFC | 12 | 74.00% | 72.25% | 72.92% | 0.8188 |
| SFC | 5 | 50.20% | 56.44% | 54.04% | 0.6025 |
|  |  |  |  |  |  |
| Combine | 14 | 79.80% | 67.44% | 72.19% | 0.7804 |

The results of using the classification models with the testing set also showed that DFC-based optimal features achieved the best performance, which was severe inconsistent with the performance using the training set. Fig. S4 shows the ROC curves of using the optimal models with the testing set.

Fig. S4 the ROC curves of using the optimal models with the testing set

In addition, these results further reveal that the optimal model developed using the DFC-based optimal features extracted from the brain regions using the Brainnetome atlas could be more powerful for the discrimination between MDD patients and the healthy people.

All the results above conclusively reveal that the classification model developed using the DFC-based optimal features extracted from the brain regions using the Brainnetome atlas template could be more powerful for the discrimination between the MDD patients and the healthy people, including the SFC-based features could probably introduce certain feature redundancy that might further impair the discriminative power of the prediction model.
